# Restless legs syndrome: abbreviated guidelines by the German sleep society and the German neurological society

**DOI:** 10.1186/s42466-024-00353-0

**Published:** 2024-11-06

**Authors:** Claudia Trenkwalder, Ambra Stefani, Cornelius G Bachmann, Christian Maihöfner, Johannes Mathis, Lucia Muntean, Julian Mollin, Joachim Paulus, Anna Heidbreder

**Affiliations:** 1 Paracelsus-Elena Klinik, Center of Parkinsonism and Movement Disorders, Klinikstraße 16, Kassel, Germany; 2 Department of Neurosurgery, University Medical Center, Goettingen, Germany; 3 Department of Neurology, Medical University of Innsbruck, Anichstr. 35, Innsbruck, Austria; 4 Department of Neurology, University Medical Center, Goettingen, Germany; 5 Somnodiagnostics, Martinistrasse 63-65, Osnabrück, Germany; 6 Clinic for Neurology, Klinikum Fürth, Jakob-Henle-Straße 1, Fürth, Germany; 7 Neurocenter Bern, Schänzlistrasse 45, Bern, Switzerland; 8 Department of Paediatrics and Adolescents’ Medicine, Klinikum Westbrandenburg, Charlottenstraße 72, Potsdam, Germany; 9 RLS e.V. - German Restless Legs Association, Schäufeleinstr. 35, Munich, Germany; 10 Clinic for Neurology, Johannes Kepler University, Linz, Austria

**Keywords:** Restless legs syndrome, Sleep, Therapy, Augmentation, Dopaminergic, Opioid, Pregnancy, Children

## Introduction

This article is an abbreviated and translated version of the guidelines on restless legs syndrome (RLS) [1] prepared for and approved by the German Society for Neurology (Deutsche Gesellschaft für Neurologie, DGN) and the German Society for Sleep Research and Sleep Medicine (Deutsche Gesellschaft für Schlafforschung und Schlafmedizin, DGSM), covering both diagnostic and therapeutic options. A complete version of these guidelines (in German) can be found on the DGN website (www.dgn.org) and the Arbeitsgemeinschaft wissenschaftlicher Medizinischer Gesellschaften (AWMF, https://register.awmf.org/de/leitlinien/detail/030-081, registry No. 030/081, first published May 2002; last update: January 30th, 2024; valid until: June 24th, 2027; last access: September 25th, 2024. The level of these guidelines is S2K.

The original multidisciplinary task force who developed these guidelines consisted of 13 voting members* (*A Heidbreder, C Trenkwalder, CG Bachmann, M Bartl, S Fulda, L Habersack, C Maihöfner, J Mathis, L Muntean, B Schneider, A Stefani, J Paulus, and P Young) and an independent moderator. This group included neurologists, sleep physicians, pediatric neurologists, and patient representatives from Germany, Austria, and Switzerland. In addition to being approved by the Guidelines Commission of the DGN and the DGSM, they were approved by the executive boards of the following societies: Deutsche Schmerzgesellschaft e. V., Deutsche Gesellschaft für Kinder- und Jugendmedizin e. V. (DGKJ), Schweizerische Neurologische Gesellschaft (SNG), Österreichische Gesellschaft für Schlafmedizin und Schlafforschung (ÖGSM), Schweizerische Gesellschaft für Schlafforschung, Schlafmedizin und Chronobiologie (SGSSC).

The original guidelines were translated into English using an artificial intelligence translation tool (DeepL https://www.deepl.com) before being edited by the authors and a native English speaker (A-M W).

The national guidelines for the diagnosis and treatment of RLS were last updated in January 2024. The objective of these guidelines is to provide comprehensive information on the classification, diagnostic procedures, differential diagnosis, and treatment of RLS. They integrate the latest scientific findings on RLS treatment and the management of complications, with specific considerations for RLS treatment during pregnancy, breastfeeding, and in children and adolescents.

## Methodological approach

The project was managed by the coordinators CT and AH. The topics were worked on by author-teams based on the current data situation and then coordinated in a first Delphi round by the guideline group.

### Key questions

The systematic search, selection, and critical appraisal of the evidence began with the formulation of key questions. For structuring purposes, the primary key questions for each subchapter were developed during a plenary meeting of the task force and were summarized and agreed upon in a subsequent meeting.

### Literature search

These S2K-level guidelines (AWMF-registry No. 030/081) are based on a review of publications (primarily reviews and meta-analyses) from the previous ten years related to the selected questions. If any of the agreed-upon questions could not be adequately answered with these publications, additional scientific papers or older relevant publications were consulted.

### Structured consensus findings

The core statements of these guidelines were evaluated according to the Oxford Centre for Evidence-based Medicine - Levels of Evidence, and the strength of recommendation was derived accordingly. A strong recommendation is indicated by the term “shall,” a recommendation by “should,” and an open recommendation by “can.”

Formal consensus-building procedures were carried out through multiple plenary meetings (on 25 June 2020, 8 September 2020, and 24 November 2020) to establish subchapters, key questions, key words, and authorship. The final manuscript was written by the lead authors, revised by all contributors, and discussed further in a series of consensus conferences (on 29 September 2021, 25 November 2021, and 21 December 2021), where recommendations were voted on using a nominal group technique.

The final manuscript was written and revised by the lead authors and sent to all authors for review and edits (including additions and modifications). Each author edited and commented on each subchapter.

None of the authors abstained due to conflicts of interest. Simultaneously, as part of a Delphi process, participants indicated their agreement or disagreement with the original background text of the guidelines [1].

### Grading of recommendations and determination of consensus strength

The strength of consensus was determined as shown in Table 1.

**Table 1:** Grading of recommendations and determination of consensus strength

Classification of consensus strength	Percentage of those eligible to vote
Strong consensus	> 95% of those eligible to vote
Consensus	> 75–95% of those eligible to vote
Majority agreement	> 50–75% of those eligible to vote
No majority approval	< 50% of those eligible to vote

Recommendation category	**Definition**
Strong recommendation	Shall / shall not
Recommendation	Should /should not
No majority recommendation	Can be supported / may be supported

### External review

After the authors completed the guidelines, they were submitted to the named societies or their executive boards for review and approval.

## What is new?

### Diagnosis/additional investigations

The essential diagnostic criteria for the diagnosis of restless legs syndrome (RLS) were developed by the International Restless Legs Syndrome Study Group (IRLSSG) in 2014 and agreed upon in a consensus conference. Unlike in the previous diagnostic criteria, they now also explicitly include the exclusion of differential diagnoses, thus enabling a better differentiation from other disorders with similar symptoms. In addition, specifications for the clinical course and significance of the symptoms were formulated and added, which allows a clinical classification of the severity and course of the symptoms.

The diagnosis is clinically established when the diagnostic criteria (see definition and classification) are met.

Polysomnography is not mandatory for RLS diagnosis, but it can, however, be helpful, since the detection of increased periodic leg movements in polysomnography is a supporting criterion in the diagnosis of RLS. Polysomnography should be performed to rule out sleep-related breathing disorders and, under certain conditions, also when parasomnias, hypersomnias, or insomnia are present.

Iron metabolism (serum ferritin, transferrin saturation, iron, and iron-binding capacity) and blood count should be determined in all patients with RLS, both at the time of diagnosis and at the start of therapy and whenever there is a worsening of RLS symptoms.

Generally, there are no validated questionnaires that can be recommended for the sole diagnosis of RLS. However, screening questionnaires can be used as part of a multi-stage procedure, provided that a personal interview is part of the diagnostic process.

To assess RLS severity and characterize RLS, three assessments have been validated in the German-speaking area: the International RLS Study Group Severity Scale (International RLS Severity Scale – IRLS), the RLS-6 scales and the Augmentation Severity Rating Scale (ASRS). The use of these scales is only useful for quantifying symptoms in patients with an established diagnosis of RLS and should be used to assess the course of RLS in patients.

The previous distinction between primary and secondary RLS has been replaced by a concept in which RLS arises from interactions between genetic, socioeconomic, and environmental factors, as well as comorbidities. For this reason, the term “secondary RLS” should no longer be used.

### Therapy

The decision to indicate drug therapy should be determined by the extent to which RLS symptoms reduce quality of life and sleep quality. The initiation of chronic/continuous drug therapy should be delayed as long as possible. Medications that may exacerbate RLS should be identified and modified accordingly. All comorbidities and diseases should be diagnosed and treated early. RLS differential diagnoses should be recognized and specifically treated.

Oral iron supplementation should be given for mild RLS and ferritin ≤ 75 µg/L. For moderate to severe RLS or when oral iron is contraindicated/not tolerated, intravenous treatment should be considered. If insufficient symptom reduction is achieved or the conditions for iron supplementation are not met, non-ergot (NE) dopamine agonists are approved and efficacious for the first-line treatment for RLS in Germany, Austria, and Switzerland. To prevent augmentation, the dosage of dopamine agonists should be kept as low as possible, and always within the approved dose for RLS. Only one dopaminergic substance should be used.

Therapy for RLS should initially be with a NE dopamine agonist or with a gabapentinoid (gabapentin/pregabalin) (off-label use). Levodopa should not be used for continuous treatment, but only intermittently and/or for diagnostic purposes at a maximum dose of 100 mg. In the event of augmentation or treatment failure in moderate to severe RLS with the above medication, second-line medications such as opioids like oxycodone/naloxone ret. or sustained-release preparations (off-label use) can be used.

Cannabinoids, magnesium, and benzodiazepines cannot be recommended for the treatment of RLS. Non-pharmacological treatment options can be used alone or in addition to medication. Evidence is available for transcutaneous spinal direct current stimulation (tsDCS), movement training (e.g. stationary bike during dialysis, yoga) and infrared light therapy. The use of acupuncture, pneumatic compression, endovascular laser ablation, cryotherapy, and phytotherapy cannot be recommended at present due to the lack of data.

For the diagnosis of RLS, all diagnostic criteria must be met in pregnancy as well as in children and adolescents. In pregnant women with RLS, iron replacement should be given if the ferritin level is ≤ 75 µg/L. No medication used to treat RLS in adults is approved for use in children and adolescents.

Iron replacement is the first-line drug therapy for children and adolescents.

**Guidelines and recommendations**

A strong consensus was reached for all recommendations in each sub-chapter.

## Definition and classification

Key questions:

1. What are the current diagnostic criteria?
2. How can people with limited communication skills be diagnosed with RLS?
3. What are the objective findings of RLS?
4. Is there an atypical RLS?
5. Who can diagnose RLS?
6. How is RLS diagnosed?
7. What are RLS mimics and how are they ruled out?
8. Is polysomnography necessary, sufficient or helpful in diagnosing RLS?
9. Which laboratory diagnostics are necessary, sufficient or helpful for the diagnosis of RLS?
10. Which assessments (incl. questionnaires) are validated for RLS?

A positive diagnosis of RLS requires that all five of the following essential diagnostic criteria be met [2]:

1. An urge to move the legs usually but not always accompanied by or felt to be caused by uncomfortable and unpleasant sensations in the legs.
2. The urge to move the legs and any accompanying unpleasant sensations begin or worsen during periods of rest or inactivity such as lying down or sitting.
3. The urge to move the legs and any accompanying unpleasant sensations are partially or totally relieved by movement, such as walking or stretching, at least as long as the activity continues.
4. The urge to move the legs and any accompanying unpleasant sensations during rest or inactivity only occur or are worse in the evening or night than during the day.
5. The occurrences of the above features are not solely accounted for as symptoms primary to another medical or behavioural condition (e.g. myalgia, venous stasis, leg oedema, arthritis, leg cramps, positional discomfort, habitual foot tapping).

The current RLS diagnostic criteria, established by consensus in 2014 by the International RLS Study Group (IRLSSG) [2], introduced a key change from previous criteria: a fifth criterion now requires the exclusion of differential diagnoses, thereby better differentiating RLS from mimics. Conditions that mimic RLS can also be present as comorbidities in these patients. In such cases, RLS symptoms should be clearly differentiated from those of the comorbid condition. The diagnostic criteria include specifications for determining the clinical course (chronic/persistent or intermittent) and defining the clinical significance of RLS symptoms.

People with impaired communication skills or with dementia are assessed using the same diagnostic criteria. Specific criteria for these populations have yet to be developed.

Some patients report symptoms that do not correspond to the classic characteristics of RLS. These patients are classified as having atypical RLS. However, even in atypical forms, all diagnostic criteria must be met for an RLS diagnosis to be confirmed.

There are no validated questionnaires that can be recommended as stand-alone instruments for diagnosing RLS. However, screening questionnaires can be used as part of a multi-stage procedure provided that a personal interview is included. Three assessments have been validated in German to assess and characterize RLS severity: the International RLS Severity Scale (IRLS) [3], the RLS-6 scale [4], and the Augmentation Severity Rating Scale (ASRS) [5]. These scales are only useful for quantifying symptoms in patients who have already received an RLS diagnosis and should be used to assess the severity or progression of RLS symptoms.

The IRLSSG recommends assessing iron metabolism (serum ferritin, transferrin saturation, iron, and iron binding capacity) and blood count for all RLS patients at the time of diagnosis, at the start of treatment, and if RLS symptoms worsen during the course of the disease.

As stated above, RLS diagnosis is clinical, and instrumental assessments are not required. If polysomnography is performed it detects periodic leg movements of sleep (PLMS; with an index > 15/h) in > 80% of patients with RLS. PLMS support RLS diagnosis. While routine polysomnography should not be performed in RLS patients, it is necessary in some cases, such as when other sleep disorders—e.g., sleep-related breathing disorders, parasomnias, or hypersomnias—need to be excluded.

The classification of RLS into primary and secondary forms has been superseded by a more nuanced understanding of the disease: RLS is now recognized as a multifactorial disease involving interactions between genetic, socio-economic, and environmental factors as well as comorbidities that contribute to the clinical picture. Therefore, the term “secondary RLS” should no longer be used [6].

## Pathophysiology

Key questions:

1. What are the current data on the pathophysiology of RLS?
2. What genetic factors are known for RLS?
3. What role does iron metabolism play?
4. What role does dopamine play?
5. Are there other factors that play a role in the pathophysiology of RLS?
6. Are specific regions of the brain affected?

The exact pathophysiology of RLS is not yet fully understood but we know that it is influenced by genetic factors, dopamine, and iron metabolism. Various studies also indicate that peripheral hypoxia [7–11] may play a role in its pathophysiology.

A positive family history is common in patients with an early onset of RLS (before the age of 45) with reported prevalence rates ranging between 40% and 92% [12].

Genome-wide association studies (GWAS) have identified risk loci associated with RLS. These include six loci encompassing the genes for MEIS1, BTBD9, SCOR1/MAP2K4, PTPRD, and TOX3, which are associated with an increased risk of RLS. Thirteen other risk loci play a role in neuronal development, axon formation, and synapse formation, and are associated with neuronal differentiation [13]. One GWAS identified a new association involved in one pathway of iron metabolism as well as other important pathways [13].

In RLS, iron deficiency affects the central nervous system (CNS). This has been confirmed by studies documenting reduced ferritin and increased transferrin levels in the cerebrospinal fluid (CSF) of RLS patients, as well as by imaging studies showing reduced iron levels, particularly in the substantia nigra and other brain areas [14, 15]. The role of dopamine in the pathogenesis of RLS is proven by the effectiveness of dopaminergic therapy, even though there is no actual lack of dopamine. Iron deficiency is assumed to result from a disorder of iron regulation in the CNS [14]. Additionally, recent findings show mitochondrial iron deficiency is associated with mitochondrial dysfunction [15].

Imaging studies using SPECT and positron emission tomography (PET) show decreased striatal dopamine D2 receptor binding [16], indicating increased synaptic dopamine and increased tyrosine hydroxylase in the substantia nigra in RLS patients [17]. Together, these data indicate a presynaptic hyperdopaminergic and postsynaptic hypodopaminergic state in RLS [18].

Several studies indicate that hypoxia plays a role in the pathogenesis of RLS, through peripheral hypoxia [7] and activation of hypoxia-inducing factors (e.g. HIF-alpha) [11]. However, imaging studies have not consistently shown structural CNS changes. Most changes are located in the limbic and nociceptive network [19, 20], or in the thalamus for sensorimotor dysfunction [21–23].

## Comorbid RLS, differential diagnosis

Key questions:

1. Which causes of comorbid forms of RLS are important?
2. What are the clinically relevant differential diagnoses of RLS?
3. What are the ‘red flags’ for which other or additional differential diagnoses (mimics) should also be considered?
4. Which diagnostic procedures are necessary to adequately identify both the comorbid forms and the differential diagnoses of RLS?
5. Which comorbidities or co-medications can worsen RLS?

The distinction between comorbid RLS and RLS mimics is complicated by overlapping etiologies. There is a lack of scientific evidence to determine whether certain diseases cause RLS or are simply associated with RLS due to common etiological factors. It remains unclear whether comorbid conditions induce an “RLS-like syndrome” or if they are only comorbid disorders.

For the differential diagnosis of RLS and neuropathy, the usual neurophysiological examinations such as neurography and electromyography are used in addition to a detailed history of the patient (according to diagnostic criteria). All comorbid factors and diseases should be diagnosed and treated as early as possible (Table 2).

**Table 2:** RLS in neurological and other diseases

RLS in neurological diseases	
RLS and Parkinson’s disease	Sufficient studies and meta-analyses now show that the overall prevalence of RLS is increased in idiopathic Parkinson’s disease (IPD) [24–26]
RLS and atypical Parkinson syndromes	For multisystem atrophy (MSA), different prevalences of RLS are reported—between 5% and 28% in the studies published to date [24]. Progressive supranuclear palsy (PSP) also shows an increased prevalence of RLS, whereas no increased prevalence has yet been shown for corticobasal degeneration [24].
RLS and essential tremor	The prevalence is reported to be up to 33% [24].
RLS and hereditary ataxias	Various studies have shown an increased prevalence of RLS for Friedreich’s ataxia, spinocerebellar ataxia (SCA) types 3 (Machado-Josef ataxia), 6, and 7 [24].
Multiple sclerosis and other spinal affections	The prevalence of RLS in multiple sclerosis is reported to be between 13 and 65%. It is particularly high in the presence of spinal multiple sclerosis foci [27].
Migraine and RLS	Systematic reviews have calculated an average prevalence between 15 and 20% [28].
Narcolepsy	Narcolepsy patients also suffer more frequently from RLS [29].

RLS for internal and other diseases	
Chronic pain	Approximately one-third of patients with chronic pain and fibromyalgia have RLS [30]. Patients with myalgia describe RLS-like symptoms, which often do not fulfill all RLS diagnostic criteria.
Thyroid diseases	It is suspected that thyroid dysfunction, especially hyperthyroidism but also hypothyroidism following treatment for Hashimoto’s thyroiditis, can aggravate RLS symptoms but do not directly cause RLS [31].
Chronic Obstructive Pulmonary Disease (COPD) and Obstructive Sleep Apnea Syndrome (OSAS)	The prevalence of RLS is increased in patients with COPD and OSAS [32].
Cardiovascular and cerebrovascular diseases	Several, but not all, epidemiological studies have shown an association between RLS and arterial hypertension or cardiovascular and cerebrovascular events [6, 33].
Skin diseases	In psoriasis, the prevalence of RLS is 15% according to standard diagnostic criteria, while in atopic dermatitis, one study reported a prevalence as high as 40% [34].
Irritable bowel syndrome and irritable bladder	Up to a quarter of patients with irritable bowel syndrome fulfill the RLS essential diagnostic criteria. RLS prevalence is higher (30%) in both coeliac disease and Crohn’s disease. There is a bidirectional relationship between RLS and irritable bowel syndrome resulting in frequent co-occurrence [35].
Vitamin D deficiency	Some studies have reported reduced vitamin D levels in RLS patients compared to healthy controls. However, replenishment of vitamin D levels did not influence RLS symptoms in any of the studies [36].

An important differential diagnosis for RLS is polyneuropathy (PNP). In PNP, paresthesia typically begins symmetrically, resembling the sensation of wearing socks, whereas RLS symptoms usually begin deep in the lower legs. PNP are often described as formication, numbness, or burning and are localized superficially in the skin. In contrast, RLS symptoms are much more likely to be localized deep within the muscles, bones, or joints. A combination of both RLS and polyneuropathy is not rare, but polyneuropathy may be a risk factor for developing RLS.

Although nighttime sensory symptoms in bed also occur with PNP and cause great discomfort, the symptoms are usually also present during the day, and unlike RLS symptoms, exercise does not provide complete and lasting relief. Clinical examination of PNP reveals a reduced sense of vibration and absence of Achilles tendon reflexes. The diagnosis is confirmed by neurophysiological methods.

Other RLS differential diagnoses should also be recognized and specifically treated (see Table 3).

**Table 3:** RLS mimics and differential diagnosis

RLS mimics/differential diagnosis	
Positional discomfort	Unpleasant pulling pain in the joints after prolonged sitting in the same position but not lying down. Relief is provided by simply changing position.
Narrow spinal canal, radiculopathies	Strain-dependent back pain, sometimes with radiation, mainly when walking downhill or lying on the back. Relief when sitting down or adopting a stooped posture.
Intermittent claudication	Pain increases when walking and is relieved when standing still, sitting, or lying down. No urge to move, no circadian rhythm.
Varicose veins	Increase in discomfort on prolonged standing, relief on elevation, often objective findings of the chronic venous symptom complex.
Osteoarthritis/arthritis	Symptoms predominantly in the joint area, increase on exertion, no circadian rhythm.
Morton’s neuralgia / tarsal tunnel syndrome	Exercise-dependent burning pain in a circumscribed innervation area on the foot with pseudoneuroma of a digital nerve, rarely bilateral. No circadian rhythm and no improvement with movement.
Voluntary foot movements (foot tapping/ rocking)	Rhythmic rocking with the feet when bored or anxious. No sensory complaints, no urge to move. No circadian symptoms.
Sleeping stereotypes	Rhythmic movements of the head (jactatio capitis) or body (jactatio corporis) while awake immediately before falling asleep, without sensory complaints, but with a strong urge to perform the movements.
Foot tremor	Rhythmic movements of the feet, unilateral, bilateral, or side alternating.
Tic diseases	Similar to RLS, the involuntary stereotypies in tic disorders can only be voluntarily suppressed for a limited time. An increasing inner tension ultimately forces the movement to be allowed, which leads to relief. Increase in tension and stress and decrease in a relaxed environment. No circadian dependence.
Hypotensive akathisia	In patients with orthostatic hypotension, inner restlessness occurs while sitting, and accentuation in the legs is possible. Relief by walking around. No complaints when lying down, no circadian rhythm.
Orthostatic tremor	Subjective “dizziness” or unsteadiness only when standing still for a long time, and disappears when walking, sitting, or lying down. No sensitive sensations, no circadian rhythm.
Myoclonus of falling asleep	Isolated twitching of the whole body with emphasis on the legs, immediately before falling asleep. Particularly frequent in uncomfortable positions, e.g. when sitting, and when taking stimulants (caffeine, Ritalin). The twitching may be accompanied by short-lasting sensitive sensations, and no urge to move. It is important to distinguish from periodic leg movements during wakefulness (PLMW).
Propriospinal myoclonus	Similar to sleep onset myoclonus but repetitive and trunk-emphasized (e.g. jackknife phenomenon). In addition to spinal lesions or impaired supraspinal inhibition, a functional cause has been suspected in some cases.
Fasciculations, myokymia	Benign fasciculations without muscle weakness are common, especially in the calf muscles. Myokymia occurs focally, especially periocularly and sporadically during drowsiness. Localization of symptoms is more clearly defined than RLS.
Painful legs and moving toes	Permanent, irregular rocking movements of the toes, more rarely of the fingers around 1–2 Hz, which are not accompanied by an urge to move, tend to increase with movement and can only be suppressed arbitrarily for a short time. No sensory complaints, no circadian rhythm.
Nocturnal calf cramps	Typically, a sudden contraction of a single muscle, usually the calf or foot muscles, often during sleep. Secondary spread to other muscles is common. Relief by stretching or walking around.
Akathisia	Combination of restlessness of movement—especially of the legs—in conjunction with inner restlessness. Those affected find it difficult to maintain a calm position when sitting or standing and either have to stand up, walk around, constantly change legs or constantly move their legs while sitting, or rock their upper body forwards and backwards. No discomfort, no circadian rhythm, and usually no sleep disturbances.
Attention deficit hyperactivity disorder (ADHD)	Urge to move with inner restlessness, accompanied by difficulty falling asleep and frequent PLMS. No sensory complaints. However, transitions to RLS and a genetic relationship between the two diseases are possible.
Restless genital syndrome	The unpleasant sensations, which are not localized in the legs but in the genitals, also occur mainly at rest towards the evening and are relieved by activity (including sexual activity). No feelings of pleasure. Often associated with typical RLS symptoms.

## RLS Therapy

Key questions:

1. Why should RLS be treated?
2. When should RLS be treated?
3. What drug therapy options are available?
4. Which non-medication therapy options are possible and useful and are available?

Due to delayed and incorrect diagnoses and therapies, as well as treatment complications such as augmentation, RLS imposes a high socioeconomic burden on Western European healthcare systems (Trenkwalder, 2021 #8909). Treatment initiation is determined by a patient’s level of suffering. Furthermore, an increasing number of studies suggest an association between RLS and cardiovascular diseases [37–39], indicating that effective treatment of RLS could reduce putative cardiovascular risk factors, such as nocturnal blood pressure elevation or increased heart rate [38, 39].

### Recommendations on when to treat RLS

The most decisive criterion for treating RLS is impaired quality of life due to symptoms such as the urge to move, pain, insomnia, and daytime tiredness. In patients with RLS and comorbid diseases—cardiovascular, psychiatric, or obstructive sleep apnea—treatment of these comorbidities should be carefully considered as the first option if possible. Once all RLS diagnostic criteria are met and differential diagnoses are recognized and adequately treated, either intermittent or continuous therapy should be initiated at the lowest possible dose. Continuous pharmacological therapy should be prescribed, also at the lowest possible dose, and should be delayed for as long as possible. Concomitant medications that exacerbate RLS should be changed or stopped. See Table 4 for an overview.

### Substances with sufficient evidence for effectiveness

#### Iron

A meta-analysis of iron treatment in patients with RLS reports a low to moderate superiority of iron treatment compared to placebo, with a significant improvement in RLS severity at moderate evidence levels, even with normal ferritin serum concentration at study baseline [40]. According to an expert consensus for German-speaking countries, serum ferritin < 75 µg/L or a transferrin saturation ≤ 20% are indications for oral or intravenous iron treatment. For oral administration, iron III sulfate is recommended [41]. Intravenous iron is recommended when oral iron has failed to increase serum ferritin levels or if there are contraindications/intolerances to oral iron. The most common side effects leading to the discontinuation of oral iron therapy are gastrointestinal issues, such as constipation and nausea.

##### Intravenous Iron

**Recommendation:** In addition to intolerance to oral iron, intravenous iron therapy with ferric carboxymaltose (FCM) should be considered for the treatment of moderate to severe RLS. To avoid iron overload and accumulation in the liver, serum ferritin levels should not exceed 300 µg/L, and transferrin saturation not exceed 40% [41].

A verification of iron metabolism parameters, including serum ferritin, should be undertaken 12 weeks after intravenous administration of FCM. Data from a randomized clinical trial of FCM in RLS reports that RLS symptoms significantly improve at 12 weeks, but not earlier [42]. Therefore, a clinical evaluation should also be performed.

Given the risk of anaphylactic reactions with high molecular weight iron dextrans, these formulations present an unacceptable risk. They are no longer approved in many countries, including Germany, and should not be considered.

#### Dopaminergic agents

##### Levodopa

Several randomized, double-blind studies report on the efficacy of levodopa for the treatment of RLS. Levodopa/benserazide is approved for RLS treatment in Germany, Austria, and Switzerland.

**Recommendation:** Due to the high augmentation rates observed with daily levodopa doses, particularly at doses ≥ 200 mg, its use as continuous therapy is no longer recommended [43]. Due to the risk of excessive self-medication, levodopa should only be administered for diagnostic purposes [44] or as intermittent therapy at a dose of 100 mg.

#### Non-ergot dopamine agonists

In Germany, the first-line treatments for RLS are the standard preparations of the dopamine agonists: pramipexole, ropinirole, and the rotigotine transdermal patch. The latter is continuously effective over 24 h, thereby providing relief for daytime RLS symptoms. Alternatively, the sustained-release forms of pramipexole or ropinirole can be prescribed (not approved in the EU for the treatment of RLS, reimbursement may be refused by medical insurance companies). Dopamine agonists should be prescribed at the lowest effective dose and then gradually titrated for optimal symptom relief. However, the highest recommended dose for RLS (see below) should not be exceeded.

Typical side effects of dopamine agonists, particularly during the first weeks of treatment, are oedema, nausea, orthostatic dysregulation, and dizziness [45]. If severe gastrointestinal side effects persist despite antiemetic medication, the dopamine agonist should be discontinued or switched.

Before treatment initiation, patients should be informed of the possibility of rare side effects at relatively low doses of dopamine agonists: these include impulse control disorders such as increased libido, pathological shopping, gambling, and binge eating [46]. If any of these rare side effects occur, the dopamine agonist must be discontinued. Under a stable dopamine agonist dose regimen (average pramipexole equivalent dose 0.52 mg, which is already the maximum approved dosage for pramipexole in RLS), 12.4% of RLS patients have an impulse control disorder such as binge eating, excessive purchases of food or clothing, trichotillomania, or pathological gambling [47].

Additional rare side effects include increased nocturnal wakefulness, and conversely, daytime sleepiness and sleep attacks. In such cases, activities with potential risk of injury such as driving or operating machinery, must be avoided and the medication discontinued.

In general, only monotherapy with a single low-dose dopamine agonist is currently evidence-based and should be prescribed. Rotigotine patches should not be readministered after a previous allergic skin reaction. If monotherapy with a dopamine agonist is insufficient. Monotherapy with an alpha-2-delta ligand or combination therapy with a dopamine agonist or opioid can be considered. Their combination and dosage should be established individually. No specific recommendations can be given here, as there are no clinical trial data available.

##### Rotigotine

**Recommendation:** Transdermal rotigotine is approved for the treatment of RLS at doses of 1–3 mg. The recommended dose is 2 mg; an additional dose increase to 3 mg did not significantly improve RLS severity in the approval study [48]. For further data on side effects and pharmacology see [48]. The most common side effect is local skin irritation at the application sites. According to a meta-analysis of 60 studies [49] augmentation rates are lower with transdermal rotigotine (1.7%) compared with oral dopamine agonist treatment (7.2%) [49].

##### Ropinirole

**Recommendation:** Ropinirole is recommended at an initial dose of 0.25 mg, which can be increased to 2 mg depending on symptom relief and tolerability. The approved maximum dose is 4 mg [50]. According to the results of a meta-analysis, ropinirole-associated nausea occurs with a prevalence of 37% [51]. For further data on side effects and pharmacology see [51, 52].

##### Pramipexole

**Recommendation:** Pramipexole is recommended at an initial dose of 0.18 mg. It should be titrated as low as possible to prevent augmentation. According to international consensus guidelines, the maximum daily dose is 0.52 mg [50]. However, in 2014, Allen et al. [53] reported augmentation at a maximum daily dose of 0.50 mg. For further data on side effects and pharmacology see [50].

#### Alpha-2-delta ligands

In Germany and other European countries, the alpha-2-delta ligands, pregabalin, and gabapentin are not approved for the treatment of RLS but have been shown to be efficacious and recommendations are given below.

The risk of augmentation with alpha-2-delta ligands is significantly lower compared to dopaminergic drugs.

Pregabalin increases slow-wave sleep, improves sleep maintenance, and significantly reduces wake after sleep onset according to data from a randomized controlled trial comparing placebo, pregabalin, and pramipexole [53]. However, the reduction of PLMS appears to be greater with pramipexole than with pregabalin [43]. In summary, both drug classes are effective for treating sensory RLS symptoms. Furthermore, alpha-2-delta ligands are effective in consolidating sleep, whereas dopamine agonists treat PLMS more effectively [54].

Possible dose-dependent side effects of alpha-2-delta ligands, particularly affecting older people, include dizziness, peripheral oedema, gait imbalance, ataxia, drowsiness, and visual disturbances. These severe side effects are possibly responsible for the extremely high discontinuation rates compared with placebo in several studies [55–57]. In addition, the US Food and Drug Administration (FDA) warns that gabapentin and pregabalin could cause severe respiratory disorders in patients with concomitant respiratory risk factors. This must always be taken into consideration whenever alpha-2-delta ligands are prescribed or combined with opioids.

##### Gabapentin

**Recommendation:** Gabapentin is efficacious for RLS treatment at a dose of 800 mg, and in uremic RLS at a dose of 200 mg [43]. It should be titrated as low as possible up to a maximum daily dose of 1800 mg administered as divided doses over the day. Gabapentin is eliminated renally. Therefore, a dose adjustment should be considered with concomitant renal insufficiency. For further data on side effects and pharmacology see [43].

##### Pregabalin

**Recommendation:** Pregabalin is effective in moderate to severe RLS at dosages between 150 and 450 mg taken in a single dose, one to three hours before bedtime [43]. It should be initiated at a dose between 50 and 75 mg. Pregabalin is eliminated renally. Therefore, a dose adjustment should be considered with concomitant renal insufficiency. For further data on side effects and pharmacology see [43].

#### Opioids

**Recommendation:** Following the results of a large multicentre randomized controlled trial, RLS treatment with extended-release oxycodone/naloxone has been classified as effective and safe [58]. It has been approved since 2014 at an initial dosage of 5/2.5 mg at 12-hour intervals, up to a maximal dosage of 30/15 mg at 12-hour intervals as a second-line treatment for moderate to severe RLS when other therapies fail. Oxycodone/naloxone is metabolized hepatically and renally, so dose adjustment should be made in cases of renal impairment and administration is contraindicated in cases of moderate or severe hepatic impairment. For further data on side effects and pharmacology see [43, 59, 60].

Typical side effects of oxycodone are constipation, hyperhidrosis, pruritus, fatigue, and dizziness. In RLS patients with comorbid sleep apnea, dose titration should be performed carefully with respiration monitoring: opioids prescribed in higher doses may cause respiratory depression [59, 60]. Polysomnographic data are not available from the approval study for oxycodone. In addition, sleep apnea was an exclusion criterion [58].

In general, high-dose opioid treatment can be associated with opioid-induced hyperalgesia, which may require a rotation to another opioid at an equivalent dose [61]. In the above-mentioned multicentre study, no augmentation was observed, neither during the 12-week double-blind phase nor in the 40-week open study continuation phase [58]. Augmentation during opioid therapy has been reported primarily in case studies with tramadol [62], which has a serotonergic active component and, therefore, should not be used for RLS treatment. Even though the efficacy of tilidine, a mild opioid available in Germany and several European countries, has not been studied in randomized controlled trials in RLS, its prescription in RLS can be justified according to the consensus of the guidelines committee.

### The guidelines make no recommendations for the treatment of RLS with the following drugs

#### Cannabinoids

The effectiveness of phytocannabinoids tetrahydrocannabinol (THC) and cannabidiol (CBD) has yet to be studied in randomized controlled trials in RLS. Therefore, presently, no recommendations for RLS treatment with cannabinoids can be made and treatment of RLS with cannabinoids should not be undertaken [63].

#### Magnesium

Oral magnesium administration is often anecdotally recommended for the treatment of leg cramps and is, thus, suggested as a treatment for RLS. A systematic review on the efficacy of magnesium treatment in RLS based on three case series, four case reports, and one single randomized controlled trial was inconclusive [64]. Consequently, RLS should not be treated with magnesium.

#### Benzodiazepines

Carlos et al. [65] did not identify any studies assessing the efficacy and safety of benzodiazepines for treating RLS that met the standards required for inclusion in a meta-analysis according to Cochrane analysis guidelines. Based on this meta-analysis and expert consensus, we conclude that due to insufficient study data, the effectiveness of benzodiazepines in treating RLS is unknown. Consequently, RLS treatment with benzodiazepines should not be prescribed.

**Table 4:** Pharmacotherapy of RLS

Pharmacotherapy of restless legs syndrome			
Active ingredient group	Preparation	Min-Max. dosage	Notes
**First-line treatment**			
Iron supplements	Iron-III-sulfate	100 mg daily Each with 100 mg vitamin C p.o.	For mild RLS and ferritin ≤ 75 µg/L or transferrin saturation < 20%.
	Ferric carboxymaltose	1000 mg single dose intravenous	For moderate to severe RLS and ferritin < 75 and not > 300 µg/L or transferrin saturation < 20%; or intolerance or absorption disorders with oral iron administration.
Dopaminergic agents	Pramipexole	0.18-0.52 mg	Maximum dosage: 0.52 mg. Average dose in clinical trials 0.18–0.52 mg. Extended-release form is off-label
	Ropinirole	0.25-4 mg	Maximum dosage: 4 mg. Average dose used in clinical trials: 0.5-1 mg. Extended-release form is off-label.
	Rotigotine	1–3 mg	Recommended dose: 2 mg, an additional dose increase to 3 mg did not significantly improve RLS severity in the approval study.
Pregabalin and gabapentin	Gabapentin	200 mg/ up to 1800 mg in several doses throughout the day	Off-label use.
	Pregabalin	150 mg-450 mg 150-300 mg Single dose in the evening	Off-label use.
**Second-line options**			
Opioids	Oxycodone/Naloxone ret.	2 × 5/2,5 mg − 2 × 10/5 mg; max: 2 × 30/15 mg	Average effective dose: 2 × 10/5 mg, note sleep apnea syndrome, check if necessary. Side effects: nausea, constipation; allergies, headache.
**Sporadic/intermittent RLS**			
Dopaminergic agents	Levodopa/dopamine decarboxylase inhibitor (carbidopa or benserazide)	100 mg/25 mg	If possible, only 100 mg/d, high risk of augmentation, if possible, only intermittent administration or for diagnostic purposes.

### Non-pharmacological therapies: Therapies with sufficient evidence for effectiveness

#### Exercise

Since the publication of the previous German Neurological Society (DGN) guidelines on RLS, a study on patients with RLS and uremia shows that dialysis three times per week combined with the use of bed bicycles alleviates RLS symptoms [43, 66]. Therefore, dialysis with bed bikes is a non-pharmacological recommendation for uremic patients with RLS.

**Recommendation:** Sufficient evidence indicates that yoga improves sleep quality and reduces symptom severity in RLS patients [67, 68]. However, it remains unclear which specific aspects of exercise training alleviate RLS symptoms or the optimal time point for exercise training. Intense exercise in the afternoon or evening seems to worsen RLS symptoms at night [43, 69]. Further studies are necessary to clarify these questions.

#### Transcutaneous spinal direct current stimulation (tsDCS)

One double-blind and two single-blind controlled randomized trials conducted by two independent groups show RLS symptoms are relieved with anodal transcutaneous spinal direct current stimulation (tsDCS). tsDCS reduces spinal excitability and is considered a safe method [70, 71].

**Recommendation:** Although further investigations on the clinical applicability and effectiveness of tsDCS over the long term are needed, it is recommended as a non-drug therapy option. Currently, it lacks approval as a treatment for RLS and is not readily available.

#### Infrared light therapy

A single-blind, sham-controlled, randomized study assessed the efficacy of infrared light therapy at acupuncture points in RLS patients with renal insufficiency requiring dialysis (*n* = 60). Compared to sham treatment, infrared light therapy significantly reduces the IRLSS severity scale score [72].

**Recommendation:** Infrared light therapy is recommended as a non-pharmacological therapeutic option for RLS, although further trials are urgently needed to prove its efficacy. Currently, it lacks approval as a treatment for RLS and is not readily available.

#### Sleep hygiene

Although there is currently no evidence for the effectiveness of sleep hygiene in RLS, it is generally considered useful.

### Insufficient evidence to make recommendations

The following treatment strategies have been studied in patients with RLS, but there is insufficient evidence for recommendations, therefore, they are only listed here, and references are provided for further details:

Endovascular laser ablation (ELA) [73, 74].

Cryotherapy of the whole body [75].

Pneumatic compression of the legs [43, 73, 76].

Acupuncture: Metanalysis of the Cochrane Collaboration [77]: negative, single more recent study positive [78]. At present, the studies are too heterogeneous, and there is insufficient evidence of the effectiveness of ear acupuncture.

Phytotherapy with peony root (Paeoniae Radix) [79].

## Augmentation

Key questions:

1. How is augmentation diagnosed?
2. How should augmentation be treated?
3. Can augmentation be avoided?

Augmentation is currently the most frequent and clinically relevant side effect of dopaminergic therapy for RLS. Patients should be routinely monitored for augmentation during treatment with dopaminergic agents.

### Recommendation for diagnosing augmentation

Augmentation is diagnosed clinically/anamnestically. The following criteria should be present for the diagnosis of augmentation:

- Advancement of the onset of RLS symptoms by approximately 2 h.
- Spread of symptoms to other parts of the body.
- Increase in the intensity of symptoms compared to when therapy started.
- Decrease in the effect of the dopaminergic medication.
- Mimics and opioid-induced hyperalgesia should be excluded.

### Recommendation for avoiding augmentation

To avoid augmentation, RLS should be treated primarily with an alpha-2-delta ligand or the dose of any dopaminergic medication should be kept as low as possible.

### Recommendation for treating augmentation

If augmentation is present, it should be treated in a stepwise approach by following these measures:

- Control of iron metabolism: if serum ferritin value ≤ 75 µg/L or transferrin saturation < 20%, iron should be substituted (intravenous or orally depending on the clinical picture).
- The dose of dopaminergic medication should primarily be reduced to the maximum approved dosage over 24 h; the medication can be divided into several doses (no studies available).
- In the case of augmentation with levodopa, a switch to dopamine agonists should be made. However, there is also a risk of augmentation developing again.
- In the case of augmentation with dopamine agonists, a switch should be made to a long-acting medication (e.g. rotigotine patch) or a combination with an opiate.
- Opiates can be used to reduce the total dose of dopaminergic agents.

In many patients who have been taking dopaminergic medication for a long time, the dose must be reduced very slowly to avoid dopamine agonist withdrawal syndrome.

## RLS in childhood and adolescence

Key questions:

1. How is the diagnosis of RLS in childhood and adolescence made?
2. Which therapeutic options are available for the treatment of RLS in childhood and adolescence?

In childhood and adolescence, RLS occurs with a prevalence of 1.9–3.6% (England, Turkey, USA) without differences between the sexes [80, 81]. Adults often report that their symptoms began during adolescence [82, 83]. There appears to be an increased incidence in children and adolescents who have at least one parent with RLS [80, 84]. Despite meeting all diagnostic criteria, RLS is rarely diagnosed in children and adolescents. Symptoms affect behavior, cognition, sleep, and school performance [85]. 

### Diagnosis

The diagnostic criteria in children and adolescents correspond to the diagnostic criteria for RLS (see above) [2, 85]. The urge to move and/or the sensory disturbances should be described in the pediatric patient’s own words. Care should be taken to use age- and developmentally-appropriate wording [85].

The following criteria support the diagnosis in children and adolescents [85]:

- Polysomnographic findings with periodic limb movements in sleep (PLMS) > 5/hour;
- Positive family history of RLS in a first-degree relative;
- Positive family history of PLMS > 5/hour;
- Positive family history of periodic limb movement Disorder (PLMD) in a first-degree relative.

For children and adolescents with developmental neurological abnormalities, the suggested clinical immobilization test (SCIT) is recommended [86]. A polysomnography (PSG; preferably two nights) should be performed to differentiate RLS from other sleep-related movement disorders (PLMS, PLMD; restless sleep disorder) [80, 83–85, 87]. Differential diagnoses in childhood and adolescence include attention deficit hyperactivity disorder (ADHD), position-dependent paraesthesia, muscle pain, strains or sprains, bruises, ‘growing pains’, unclear pain conditions, or dermatitis [85].

### Therapy

#### Pharmacological treatment

In children and adolescents, pharmacological therapy for RLS is used off-label, as medications that are available to treat RLS in adults are not authorized in this age group. Once the diagnosis is confirmed and the patient is experiencing clinically significant symptoms, pharmacological therapy can be considered in combination with non-pharmacological treatment [88].

**Recommendation:** The first-line pharmacological therapy for RLS in children and adolescents is iron supplementation [88]. In the presence of RLS symptoms and a serum ferritin concentration ≤ 75 µg/L, iron therapy should be prescribed for 3 months [89–94]. Oral administration of iron at 3 mg/kg body weight (bw) per day should begin with a starting dose of 1 mg/kg bw, which should be increased by 1 mg/kg bw every 3–5 days until the target dose is reached.

Intravenous iron substitution (3–6 mg/kg bw, max. 120 mg) can be administered to children and adolescents as inpatients under supervision and the following conditions:

1. Previous oral iron therapy (over 3 months) did not provide adequate benefit.
2. Oral iron therapy was discontinued due to side effects.
3. Oral iron therapy did not result in a significant increase in serum ferritin levels.

Intravenous therapy can be used without first trying oral iron therapy if there are significant concomitant diseases that impede iron absorption [41].

Levodopa has been investigated in pediatrics in a double-blind study as an effective monotherapy for RLS in children aged 7–12 years [95]. However, due to the high augmentation rate described in adults and limited data in pediatrics, continuous use of levodopa is not recommended.

Transdermal rotigotine is effective in adolescents aged between 13 and 17 years at a dose of 3 mg/24 h, as shown in a prospective multicentre study [96].

Due to insufficient data and studies in children and adolescents, no recommendations can be made for the treatment of other drugs used in adults.

### Therapy

#### Non-pharmacological treatment

**Recommendation:** Due to insufficient evidence, no recommendations can be made on non-pharmacological treatment of RLS in children and adolescents. It is assumed that regular, light physical activity, as described for adults, alleviates symptoms [69, 88]. In the presence of sleep disorders, good sleep hygiene is generally considered sensible [88].

Potential factors that aggravate or trigger RLS should be excluded and, if possible, treated [97]. Selective serotonin reuptake inhibitors, tricyclic antidepressants, metoclopramide, and diphenhydramine can exacerbate RLS symptoms. For stimulants such as coffee, alcohol, or nicotine, there is insufficient evidence that they worsen or alleviate symptoms [98, 99].

## RLS in pregnancy

Key questions:

1. How is RLS diagnosed during pregnancy?
2. What treatment options are possible for treatment of RLS during pregnancy?
  - Non-pharmacological treatment.
  - Pharmacological therapy.

RLS occurs in 15-38.8% of pregnant women, mostly in the third trimester [100, 101]. Shortly after birth, symptoms decrease significantly [102].

**Recommendation:** The diagnostic criteria for pregnancy-associated RLS correspond to the general diagnostic criteria for RLS [2, 102]. For the treatment of RLS during pregnancy, potential factors that can increase RLS should be excluded and treated if possible (these include other sleep-related illnesses and medications). If antidepressant treatment is necessary, SSRIs should be avoided. Bupropion can be given from the second trimester and after detailed counseling.

### Therapy

#### Non-pharmacological treatment

Non-pharmacological treatment such as moderate physical activity, yoga, and massage can be recommended.

#### Pharmacological treatment

**Recommendation:** Pregnant women with RLS should be prescribed iron supplementation when ferritin is ≤ 75 µg/L [41, 102]. Whether iron should be administered orally or intravenously has yet to be investigated. If iron supplementation is required before the second trimester, it should be given orally.

Ferric carboxymaltose is the preferred intravenous treatment for iron deficiency anemia in pregnancy, suitable for use from the second trimester onwards [103].

Levodopa/carbidopa can be used for the treatment of refractory RLS in pregnancy (100/25 mg to 200/50 mg standard or delayed at night or in the evening). The daily dose of levodopa should not exceed 200 mg [102]. During pregnancy, levodopa should not be administered in combination with benserazide due to the risk of embryotoxic side effects. Dopamine agonists should be avoided during pregnancy.

Low-dose oxycodone/naloxone (5–20 mg/day) can be administered for the treatment of very severe, refractory RLS during pregnancy. Low-dose clonazepam can be administered for otherwise refractory RLS in the second and third trimesters (0.5-1 mg at night). Zolpidem/zopiclone and other benzodiazepine receptor agonists should not be used; this also applies to gabapentin and pregabalin. Gabapentin has shown toxic effects on synaptogenesis in animal studies [104].

## Coordination of care

Key questions:

1. Is there coordinated care for RLS patients?
2. What primary and secondary care would be desirable?
3. What steps are necessary to optimise cross-sectoral care?

Systematically collected scientific data on the care of patients with RLS in different care areas is not yet available. Clinical experience and epidemiological studies on prevalence suggest, however, that primary care in the outpatient setting is mostly provided by general practitioners.

**Recommendation:** When RLS is first diagnosed, a specialist in neurology should be consulted. If the first diagnosis is made by a sleep physician, the family doctor should carry out further laboratory diagnostics (see section on diagnosis) and decide whether a neurological assessment is necessary.

### What primary and secondary care is desirable?

Given the high prevalence of RLS and its frequent association with various neurological and internal diseases, structured and coordinated care is essential. Initial contact should, therefore, be made with the general practitioner, who can then facilitate referral to a specialist (neurology/sleep medicine/pediatrics/neuropediatrics/psychiatry). Systematically collected scientific data on the care of patients with RLS in different care settings are not yet available.

Illustrations of the services provided for inpatients using the Diagnosis Related Groups (DRG)-system are necessary to ensure adequate quality of care. To date, the majority of diagnoses and treatment of RLS patients has taken place in an outpatient setting.

Treatment complications, such as refractory symptoms, pronounced augmentation, comorbidities, multimorbidity, and other sleep disorders can make effective care difficult in the outpatient setting. The treatment algorithm should be adjusted to accommodate these factors.

Special attention is needed for symptoms that are refractory to different treatments, as well as in patients with comorbidities (such as severe OSAS with inadequate treatment) and severe augmentation due to iatrogenic intoxication with dopaminergic substances. Here, medication can only be discontinued under close clinical supervision, including monitoring of cardiovascular parameters.

## Specific statement

The current guidelines on RLS summarize the current state of knowledge and can already be supplemented by some more recent studies. Notably, for the treatment of RLS patients with iron deficiency anemia, a comparative study of oral iron and intravenous iron showed an equivalent result of both treatments on RLS symptom severity [105]. However, this study used a different intravenous iron preparation to ferric carboxymaltose, which has been investigated in all previous studies. Nevertheless, this is the only study directly comparing oral and intravenous iron preparations in RLS patients, restricted to patients with anemia. In a study of RLS patients with no anemia and where low serum ferritin was not a primary inclusion criterion, a significant improvement on the IRLS scale, but not in QoL, was observed after 6 weeks of treatment with 750 mg intravenous ferric carboxymaltose [106].

Further therapeutic approaches could be derived from a publication on the genetics of RLS [107], which reported on the identification of new pathways of RLS. These relate to GABAergic and glutamatergic receptors, for example, and could indicate therapy with lamotrigine-type antiepileptic drugs. Although the more recent AASM guidelines [108] on RLS included a recommendation to abandon dopaminergic therapy completely due to the numerous problems with augmentation, alternatives are not sufficiently indicated. Both the efficacy of alpha-2-delta ligands and the side effects at higher doses do not represent a sufficient treatment option for severely affected RLS patients; this has already been proven by many years of clinical experience, and the few studies in RLS with pregabalin are not convincing for long-term treatment. Recent developments in the US indicate switching completely to other substances if necessary.

In summary, there is currently an urgent need for new specific drugs to treat the urge to move and the sleep disorders in RLS, and the testing of these in clinical studies [109]. All existing drugs have been adopted from other areas of medicine, e.g. Parkinson’s therapy, pain therapy, or the neuropathic field as “drug repurposing”.

The need for adequate clinical care for severely affected, mostly elderly, RLS patients with comorbidities is underestimated leading to inadequate support for these patients. Management of patients experiencing significant suffering needs to be improved by all medical disciplines, especially within the resources of the health care system.

## Data Availability

A detailed listing is available at https://dgn.org/leitlinien/.
